# A process evaluation exploring the lay counsellor experience of delivering a task shared psycho-social intervention for perinatal depression in Khayelitsha, South Africa

**DOI:** 10.1186/s12888-017-1397-9

**Published:** 2017-07-01

**Authors:** Memory Munodawafa, Crick Lund, Marguerite Schneider

**Affiliations:** 1 Alan J Flisher Centre for Public Mental Health, Department of Psychiatry and Mental Health, University of Cape Town, 46 Sawkins Road, Rondebosch, Cape Town, South Africa; 2 Centre for Global Mental Health, Institute of Psychiatry, Psychology and Neurosciences, King’s College London, London, United Kingdom

**Keywords:** Community health worker, Process evaluation, Barriers, Facilitators, Task sharing, Perinatal depression

## Abstract

**Background:**

Task sharing of psycho-social interventions for perinatal depression has been shown to be feasible, acceptable and effective in low and middle-income countries. This study conducted a process evaluation exploring the perceptions of counsellors who delivered a task shared psycho-social counselling intervention for perinatal depression in Khayelitsha, Cape Town together with independent fidelity ratings.

**Methods:**

Post intervention qualitative semi-structured interviews were conducted with six counsellors from the AFrica Focus on Intervention Research for Mental health (AFFIRM-SA) randomised controlled trial on their perceptions of delivering a task shared psycho-social intervention for perinatal depression. Themes were identified using the framework approach and were coded and analysed using Nvivo v11. These interviews were supplemented with fidelity ratings for each counsellor and supervision notes.

**Results:**

Facilitating factors in the delivery of the intervention included intervention related factors such as: the content of the intervention, ongoing training and supervision, using a counselling manual, conducting counselling sessions in the local language (*isiXhosa*) and fidelity to the manual; counsellor factors included counsellors’ confidence and motivation to conduct the sessions; participant factors included older age, commitment and a desire to be helped. Barriers included contextual factors such as poverty, crime and lack of space to conduct counselling sessions and participant factors such as the nature of the participant’s problem, young age, and avoidance of contact with counsellors. Fidelity ratings and dropout rates varied substantially between counsellors.

**Conclusion:**

These findings show that a variety of intervention, counsellor, participant and contextual factors need to be considered in the delivery of task sharing counselling interventions. Careful attention needs to be paid to ongoing supervision and quality of care if lay counsellors are to deliver good quality task shared counselling interventions in under-resourced communities.

**Trial registration:**

Clinical Trials: NCT01977326, registered on 24/10/2013; Pan African Clinical Trials Registry: PACTR201403000676264, registered on 11/10/2013.

**Electronic supplementary material:**

The online version of this article (doi:10.1186/s12888-017-1397-9) contains supplementary material, which is available to authorized users.

## Background

Common perinatal mental disorders (CPMD) are highly prevalent in Low and Middle Income Countries (LAMICS) with an estimated prevalence of one in six women [1]. Untreated perinatal depression can lead to unresponsive caregiving and have severe foetal and obstetric complications such as spontaneous pre-term labour, low birth weight and adverse child development outcomes [2–4]. CPMD is also associated with poorer quality of life as a result of impairment of maternal day-to-day functioning [5], and is exacerbated by poor socio-economic circumstances such as inadequate housing, intimate partner violence and lack of social support in LAMICS [6, 7]. In South Africa an estimated 75% of people requiring mental healthcare do not receive any due to inaccessible services and staff shortages; a figure often referred to as the ‘treatment gap’ [8, 9].

A systematic review of psycho-social interventions for CPMDs delivered by non-specialists found them to be beneficial to recipients compared to recipients that did not receive any treatment at all [10]. Psycho-social intervention such as Cognitive Behavioural Therapy (CBT), Problem Solving Therapy (PST), psycho-education and Interpersonal Therapy (IPT) have been increasingly found to be feasible and effective within High Income countries and LAMICS [6, 11]. CBT is structured therapy designed to help individuals to change unhealthy thinking patterns to healthy thinking patterns [12]. PST helps individuals to explore and identify effective solutions to problems, as well as develop sustainable problem solving skills [13, 14]. IPT focuses on interpersonal relationships and how an individual can identify the root of interpersonal stress so that they can reduce triggers to their distress and come up with alternative ways to respond to situations [15].

Task sharing can be an effective way to reduce the large treatment gap through the use of lay counsellors such as Community Health Workers (CHWs) to deliver psycho-social interventions previously designated for specialists [16–19]. Task sharing is a culturally sensitive way to increase access to care within the community by CHWs who share the same language and culture [20]. Task sharing should be provided within parameters of adequate training and supervision from a specialist [16–19] which leads to capacity building and furthers motivation to continue working [21]. There is evidence of the feasibility and acceptability of task shared interventions in LAMICS; for example, group based task shared counselling for depressed HIV positive patients was found to be feasible and acceptable in South Africa [19] and CBT and IPT have been successfully delivered by CHWs in Pakistan and Uganda [22, 23].

As much as task sharing can be effective, qualitative process evaluations highlight factors that facilitate or hinder the effectiveness of such interventions [24]. The new United Kingdom (UK) Medical Research Council (MRC) framework for process evaluations looks at the relationship between three main factors of intervention delivery - *implementation* (training, resources, fidelity, dose and reach), *mechanisms* (participant responses to the intervention and how the intervention brings about changes in the participants) and *context* (external influences to the intervention) [25]. All three factors can be interrelated and could include both challenges and facilitators to the intervention, examining these three factors can therefore help to differentiate between an intervention that is faulty in its design and a well-designed intervention that is not implemented properly [26].

For task shared interventions to be sustainable, the following *implementation* factors should be taken into consideration: training, resources, fidelity, dose and reach of the intervention. Selecting respected and motivated lay counsellors and providing adequate resources such as training, a stipend, and transport together with consistent support and supervision, will encourage higher competence and reduce distress experienced by staff [27, 28].

The assessment of fidelity (the extent to which the counsellors follow the intervention protocol as intended) is necessary when counsellors use an intervention manual in order to ensure that intervention delivery is standardised with limited variation [29]. Higher fidelity to implementation can be achieved through adequate training and supervision which help sustain motivation to deliver the intervention [30]. A fidelity checklist can be used to assess whether core intervention components were included in the delivery of the intervention [31]. *Dose* (number of the intervention sessions delivered) and *reach* (actual number of participants who receive the intervention) are vital indicators of the success of an intervention as they reflect uptake of the intervention [25, 29]. Fidelity checking also assesses the quality of delivery of the intervention [32].

The MRC guidelines for conducting process evaluations refer to *mechanisms* as participant responses to the intervention [25]. Examining the mechanisms of an intervention involves exploring how the delivered intervention is received and how that brings about change through identifying clear causal pathways [25]. In the case of a psycho-social intervention this involves looking at how the intervention leads to changes in cognitive style or problem solving skills which in turn influence mood and functioning [25]. These causal pathways cannot be examined in isolation since they are linked to the *implementation* and the *context* of the intervention [25]. Examining attendance patterns of an intervention, for example, can suggests how the participants receive the intervention [29].

The environmental *context* contains barriers and facilitators that affect the intervention [25]. Issues such as crime, violence, poverty and lack of private space are common barriers to interventions [27, 33] associated with poor attendance and drop out of care.

This study was necessary in order to explore lay counsellors’ views of facilitators and barriers to their implementation of a task sharing counselling intervention for perinatal depression in a low resource context. This information is crucial for consideration when replicating task shared interventions and [25] and when scaling up interventions in developing countries [34].

## Methods

### Affirm RCT

This study is a sub-study of the AFrica Focus on Intervention Research for Mental health, South Africa (AFFIRM-SA) randomized controlled trial (RCT). Trial Registration**:** Clinical Trials (ClinicalTrials.gov): NCT01977326, registered on 24/10/2013; Pan African Clinical Trials Registry (www.pactr.org): PACTR201403000676264, registered on 11/10/2013. The AFFIRM study was an individual-level RCT with two arms. A sample of 419 depressed pregnant women was recruited from two clinics and randomly assigned to either the intervention group or the control group. The intervention group was given a series of six to eight sessions of manual based counselling sessions over a period of three to four months (approximately two sessions per month). The control arm received a monthly phone call for three months to check on well-being without providing any counselling. This paper will focus on the intervention delivered to the intervention group only.

### Intervention development and training of CHWS

As part of the AFFIRM-SA RCT, we developed a task shared psycho-social counselling intervention, together with a basic counselling and training manual for perinatal depression in Khayelitsha, Cape Town. Detailed information on the AFFIRM-SA trial and the development of the intervention is available elsewhere [11, 35, 36]. We approached a local Non-Governmental Organisation (NGO) which provided twelve CHWs to participate in the training. These CHWs had previous experience doing health promotion visits to households in the community with mothers and their children under the age of 5 years [37]. They identified children who were underweight and at risk for malnutrition, and educated their mothers on how to improve their own wellbeing and their children’s health [37]. The counsellors were trained during a five day workshop on how to implement the manual-based intervention. Six counsellors were selected after the training based on their understanding of the training material, their level of empathy and interpersonal style displayed during the role plays. These counsellors were employed to deliver the intervention in the main trial.

### The intervention

Intervention group participants received six to eight sessions (participants had an option to finish their counselling sessions after the sixth session however they could have an additional two sessions if they felt the need for additional support). The sessions were structured manual based psycho-social individual face to face counseling sessions either at the participant’s home or at the clinic. The intervention consisted of the following sessions; psycho-education on depression and psychoeducation on birth preparation; problem solving; behaviour activation; healthy thinking and lastly termination and evaluation. The intervention was based on CBT, IPT and problems solving therapy principles [35, 36]. Participants had sessions in the antenatal phase of their pregnancies and could continue sessions into the postnatal phase if sessions were not completed by the time of the baby’s birth. Referrals were made to the Department of Health psychiatric services if participants showed any suicidal ideation. Referrals were also made to the social worker at the clinic and NGOs in the community if the assistance needed was beyond the scope of the CHWs’ intervention. All counselling sessions were digitally audio recorded to facilitate fidelity monitoring.

### Supervision and ongoing training of the counsellors

The CHWs were trained, supervised and supported by a mental health counselor (MHC) with a masters in Clinical Social Work (MM) and additional support was offered twice a month by CL (Senior Clinical psychologist and principal investigator of the AFFIRM-SA trial).

Supervision consisted of two to three hours weekly group supervision and ongoing training in addition to 30 min of individual supervision every month by the MHC between May 2013 and August 2015. Supervision focused on updates on participant progress, discussion of difficult cases, follow up on previous referrals and feedback on session notes. The counsellors reflected on their feelings while they were conducting the sessions and discussed these. The MHC took field notes during supervision and counsellors were offered mental health support by external counsellors if needed. The MHC observed initial sessions, and assessed fidelity by listening to audio recordings of the sessions on a weekly basis depending on the issues raised by the counsellors. If counsellors felt uncertain about conducting a session or if the supervisor felt that most of the counsellors needed additional training based on fidelity checks, the supervisor provided ad hoc ongoing training to revise key aspects of the intervention.

### Fidelity ratings of counsellors

A fidelity checklist (see Additional file 1) was developed by the AFFIRM-SA team through linking the basic counselling skills and session guide from the AFFIRM-SA manual to a rating scale. The checklist has 10 items divided into three main sections which are: (i) the introduction to the session (ii) exploration of the topic, and lastly (iii) ending. Each item on the check list is scored by a three tiered scoring system which includes, “*not done*” = 0, “*needs improvement*” = 1, and “*well done*” = 2.

For example, a counsellor gets a rating of 2 for introducing the topic of the session and clarifying if the participant understands the instructions before moving on to the next section compared to the counsellor who gets 1 for moving onto the next session without clarifying if the participant understands.

Scores for all three sections are added per session, and a percentage is calculated by dividing the total score by 20 (since there are 10 items with a maximum score of 2 per item) and multiplying the result by 100. Total fidelity ratings are classified into four categories which are poor (0-40%), moderate (41-60%), good (61-80%) and excellent (81-100%).

### Data collection

#### Sampling and procedure

All six female CHWs from the AFFIRM-SA study were interviewed on their perceptions of delivering the intervention after the study concluded. Table 1 below highlights the main questions that were included in the interview schedule. The full interview schedule is included as Additional file 2.

**Table 1:** Counsellor Interview Questions (not the complete interview)

a. What is your reason for wanting to be a counsellor?
b. What do you think about the week of training that you got before you started counselling?
c. Can you describe your feelings and your story about how things changed from doing the training and when you actually started doing counselling sessions with participants?
d. How did you manage to do counselling sessions in the clinics? For example, speaking to the nurses, getting space to have the sessions?
e. How do you think other nurses and clinic staff accepted you at the clinic? If you could rate their acceptance of you there, between 0 and 10, what number would you give it? 0 (you weren’t accepted) -10 (you were very accepted)?
f. Before you started the sessions what were your fears?
g. How did you rate yourself as a counsellor before you started working on AFFIRM? On a scale of 0 to 10. (Please explain)
h. How would you rate yourself as a counsellor now? On a scale of 0 to 10. *(Please explain)*
i. What did you find easy when delivering the intervention? Or what made it easy to deliver the intervention?
j. What are the challenges that you faced in delivering the counselling sessions?
k. What can be done to make these challenges easier?
l. What would you say the difference is between working with younger or older clients?
m. How many of your clients had all 6 sessions?
n. What did you notice about the type of clients who were good at coming to sessions and the type of clients who didn’t come?
o. How many people stopped attending sessions?
p. How would they show you that they were no longer interested in attending the sessions?
q. How many clients did not attend any sessions? What were their reasons for non-attendance?
r. How would you explain the different session topics:
Psycho-education about depression
Problem solving
Behaviour Activation
Healthy thinking
Psycho-education for birth preparation
Termination and evaluation
s. What do you think was the most effective part of the counselling (the part that helped the mothers most)?
t. Were there particular sessions that you think were most helpful? Which *sessions were these?*
u. Was there anything particular that you did that helped the mothers to feel more comfortable in the counselling? (If they need examples: e.g. listening, not judging, giving advice, providing a safe confidential place for the mothers to talk, etc.)?
v. Were there any particular sessions, or any particular things that you did that you thought afterwards were not very helpful? If so, what were these?
w. Which was your favourite session and why?
x. Which was your worst session and why?
y. Do you have any suggestions for ways of improving the way that the 6 sessions work?

For the fidelity rating, stratified random sampling was used to select one participant from each counsellor who had received at least six intervention sessions. This resulted in a total of six participants who yielded thirty six transcripts which were analysed by MM, with four of the counsellor sessions (24 sessions) rated by a second researcher (MS).

Semi-structured interviews (SSI) were conducted with the six counsellors at the end of the intervention to explore their experience of counselling. The interview schedule used key process evaluation components based on the MRC and Steckler and colleagues frameworks as topic guides [25, 29]. The interviews were conducted in *isiXhosa* by a trained field worker. Additional information on the interviews is provided as Additional file 3. These six interviews were audio recorded then translated and transcribed into English. The session transcripts and fidelity checklist from the sample participants were used to provide data on average preliminary fidelity ratings per counsellor. In addition to the SSIs, supervision notes were used to provide information on the development of counsellor skills and confidence, implementation challenges, strategies to overcome these challenges and the number of participants per counsellor who completed sessions.

### Data analysis

A thematic framework was developed a priori based on literature reviews with broad themes such as counselling motivation, implementation facilitators and challenges, and these were integrated into the framework approach listed below. Raw data were imported into NVivo v11 software for analysis and the framework approach was used to code the data [38, 39]. The framework approach to analysis includes five stages namely familiarisation, identifying a thematic framework, indexing, charting, mapping and interpretation. The first step was familiarisation [38, 39] (going over all the transcripts to get a better understanding of the themes). The next steps included identifying the thematic framework (integrating a priori framework and additional broad themes), indexing and charting (applying the thematic framework to data and identifying links between similar groups of broad themes), mapping (grouping more diverse themes into aggregated themes) and interpretation of what these themes mean in relation to the study that was conducted [38, 39]. The fidelity checklist was only used to code data from the thirty six session transcripts (six per counsellor) to provide an average fidelity rating for each counsellor. The supervision notes provided additional themes that were identified regarding implementation challenges and counsellor growth. Results from the fidelity checklist, supervision notes and the counsellor interviews were synthesised to provide broad assessment of the intervention implementation process.

#### Reflexivity and methodological quality

Reflexivity and methodological quality of the study was assessed using the Consolidated criteria for reporting qualitative studies COREQ, (a 32 item check list used to examine methodological rigour in qualitative studies) as set out in Additional file 3 [40]. Reflexivity refers to the examination of how the researcher’s own background, perceptions and interests impact the qualitative process [41]. The researcher took several steps to mitigate against bias: (i) an independent field worker conducted the interviews in order to avoid social desirability bias if talking to the MHC and to separate the role of the interviewer and analyst; (ii) in order to reduce fidelity bias, MM conducted the analysis of counsellor transcripts and fidelity checking of transcripts, MS also rated the transcripts for fidelity separately and any disagreements on coding were resolved through discussion until a consensus was reached; and (iii) all the researchers adhered to ethical standards required in studies of this nature as indicated in Additional file 3.

### Ethical approval

All counsellors and AFFIRM-SA study participants gave written informed consent to participate in the study and to have their interviews audio recorded and findings used for publication. Ethical approval for the AFFIRM-SA study was granted through the University of Cape Town Health Sciences Human Research Ethics Committee (HREC Reference no: 226/2011 for the main trial and 842/2014 for this specific study), the Provincial Department of Health and the local Community Health Centre (CHC) head.

## Results

All six CHWs who were recruited were part of the study until the intervention concluded. The six CHWs had education levels ranging from grade 9 to grade 12 and had at least two and a half years of previous experience in the community doing health promotion. CHW ages ranged from 28 years to 46 years, with a mean age of 37.2 years (SD 7.2 years). The counsellors received transport money, a monthly stipend, and transport to inaccessible or dangerous areas. We provide information on the intervention reach, dose and fidelity ratings of the counsellors, before presenting the results from the post intervention interviews.

### Reach, dose and fidelity ratings

Table 2 below presents a profile of the counsellors together with information on reach, dose and fidelity ratings.

**Table 2:** Profile of Counsellors

Counsellor	Age	Marital Status	Education	^a^Experience before training	Fidelity rating Poor (0–40%) Moderate (41%– 60%) Good (61%–80%) Excellent (81 – 100%)	Participants with Miscarriages, still birth or baby death	Participants with no sessions	Participants who dropped out	Participants who completed	Proportion who completed (%)
Counsellor 1	46	Widow	Grade 9	2.5 years	70%	3	4	5	23/ 35	65.7
Counsellor 2	28	Married	Grade 12	4 years	60%	3	3	7	20/33	69.6
Counsellor 3	32	Single	Grade 11	2 years	62%	5	9	9	10/33	30.3
Counsellor 4	33	Single	Grade 12	2 years	55%	3	13	10	9/35	25.7
Counsellor 5	44	Single	Grade 12	5 years	65%	4	6	7	18/35	52.9
Counsellor 6	40	Widow	Grade 12	11 years	65%	4	3	9	18/34	52.9
Total						22	38	47	98	
Mean Age of participants						28.18	27.4	27.1	27.8	

^a^Health promotion experience from working in the community for the NGO

The reach (the proportion of intended participants who actually attended the sessions) [29] of the AFFIRM intervention is 156 participants out of the 209 (74.6%) recruited in the intervention arm, including those women who dropped out of the intervention without completing six sessions. Session one (psycho-education for depression) was attended the most with 156 (74.6%) participants out of the total 209. Fifty three women (25.3%) did not engage in any sessions at all - twelve of these women (5.7% of *n* = 209) due to miscarriage or stillbirth. Fifty seven women (27.2%) dropped out of care before completing all six sessions, nine of these women due to miscarriage, or still birth and one due to participant death giving a total 22 miscarriages, still births or deaths (10.5%) out of the entire intervention sample. Only 100 participants (47.8%) completed six sessions. Out of 209 participants, 56 (26.7%) women did not complete all 6 sessions. The average fidelity rating across the six counsellors was 62.8% which shows moderate to good adherence to the manual while implementing the AFFIRM intervention; however there was wide variation in the fidelity between counsellors.

#### Perception of counsellors

The results from the post intervention interviews will be presented in the form of facilitators and barriers to the implementation followed by mechanisms and context of the intervention. Quotes will be used to illustrate the themes where relevant.

### Implementation

Facilitators to the *implementation* included counsellor factors such as motivation and competence to conduct the counselling. Intervention factors included content, supervision and using the local language as the medium of training and counselling.

All six of the counsellors were motivated to become counsellors because they were empathic and altruistic. All counsellors felt that positive feedback from participants further motivated them.

*The reason I wanted to be a counsellor is because I like to communicate with people, I like to help people.* (Counsellor 6)

Half the counsellors felt that they were confident enough to begin counselling after they received their initial training while the other half felt that they had grown as counsellors due to practice and supervision.

*I did not believe in myself that I would be able to do the job, but because of the support we received from our supervisor and the counselling I did previously I was built from that… I felt really small especially because I was not educated, but experienced… now I am very competent...* (Counsellor 1)

All the counsellors had good recall of the sessions and reported that since the content of the intervention was educational and beneficial to them and the participants, it made it easier to conduct the sessions. The counsellors enjoyed and thought the most helpful sessions were session two (problem solving) and session four (healthy thinking).

*I enjoyed session two because there were lots of twists and turns there. The mother would share her first problem and you stop her so we can talk about that problem. You ask her what she has to say about her problem. Sometimes she would leave without giving you a solution, but next time she returns with a solution…The fourth session also helped them a lot. They thought of how to move on with their lives.* (Counsellor 1)

All the counsellors felt that using the local language (*isiXhosa*) made conducting the sessions easier since participants could understand better. They also all felt that the manual based style of delivery made the sessions easier and they understood the importance of adhering to the manual.

*We could not change [the sessions]. We had to do them as they were outlined because if you changed it you would make mistakes. [The manual] provided guidance…* (Counsellor 5)

Counsellors indicated that having respect and professionalism made it easier to work with the nurses in the clinics.

*As they start witnessing the beauty of your work they become more receptive. We met with the nurses and introduced ourselves to them. Thereafter we worked together and respected each other. If they needed the room we would sacrifice and conduct the session outside.* (Counsellor 1)

Barriers to implementation of the intervention included counsellor anxiety based on fear of deviating from the manual and stress of providing emotional support. In some cases counsellor fears were attributed to how the real life situation differed from the role plays used during training.

*I was worried if I would be able to follow the manual and not add my own stuff. I was worried if I would be able to help the client with the problem they had, but at the end I was able to do so much so that others would tell me which sessions they enjoyed.* (Counsellor 2)

Half the counsellors admitted to straying from the manual in order to help the participant understand some concepts outlined in the manual when needed.

*I followed the format of the sessions, but sometimes I would find that the mother does not understand. I would need to explain…* (Counsellor 4)

All counsellors reported feeling anxious at the beginning. However three counsellors (1, 2 and 4) felt they lacked confidence to begin counselling due to inadequate training while the other three counsellors (3, 5 and 6) felt confident to begin counselling due to an adequate training period.

The counsellors’ anxiety could have been exacerbated by the supervisor’s observation of sessions as indicated below;

*When I started doing counselling sessions I wished the supervisor would not be there in the room. It is your first time and the supervisor sitting there, writing down, you feel like you are making a mistake and not doing the right thing….* (Counsellor 3)

Three counsellors mentioned how providing emotional support to mothers reminded them of their own problems and the other three mentioned how counselling participants was emotionally taxing for them.

*We forgot to take care of ourselves as counsellors. We put a lot of focus on the clients. We have our own problems and need to heal first before we help other people...* (Counsellor 4)

### Mechanisms

Counsellors felt that teaching “healthy thinking” and “problem solving” skills changed participant’s behavior.

*A person would know that she has a problem, but not know the major problem. This session taught them to list their problems and know which problem is major.* (Counsellor 2)

Counsellors also felt that older people were easier to work with compared to younger ones.

*Older people know what they want. That makes it easy to work with older people than young people… A young person attends sessions once or twice and when she feels that she is fine she stops.* (Counsellor 2)

### Context

Counselling and ongoing support allowed the counsellors to cope with their roles and prevent them from feeling overwhelmed. Two counsellors made use of external counselling services by attending one session each and the remaining four spoke to the supervisor about their personal problems during the course of the trial. Two workshops with an external organisation were conducted, the first one on trauma debriefing and the second one on managing personal finances as per the counsellors’ request. The counsellors found the trauma workshop useful for coping with some of the difficult cases they encountered and the financial workshop helped them manage their personal finances.

The next section will highlight the barriers linked to the *implementation*, *mechanisms* and *context* of the intervention.

The counselors reported facing challenges negotiating the clinic environment. When asked in the interviews to retrospectively rate the clinics in terms of being welcome, (0 being not welcome and 10 welcome), the majority of the counsellors gave Clinic A ratings which ranged from 0 to 5, indicating that they did not feel welcome at the clinic when the project started, while clinic B was rated 10 by all the counsellors, showing that the counsellors felt welcome.

*Finding a venue at the clinics was hard. Sometimes we ended up doing sessions outside and that made other mothers not attend sessions because she viewed it as unimportant because you cannot talk about something serious outside.* (Counsellor 2)

All the counsellors indicated that working with participants who needed material assistance was emotionally challenging and made them feel helpless.

*A mother would give birth and there would be nothing at home…She would phone me, “Sister I am at the hospital and my baby does not have nappies or vests or anything.” And I did not have anything myself and felt bad because I said I would be her counselor, but I am going to fail at supporting her in that way.* (Counsellor 3)

All the counsellors indicated that crime in the community made it dangerous to conduct home visits as they were worried about being robbed.

*I remember I was going to visit the mother after she gave birth…On the day that I was planning to visit her, we saw skollies (thugs) when we were on the station and had to turn back.* (Counsellor 3)

At times participants were experiencing difficult circumstances in their lives which made regular attendance and behavior change difficult.

*Most of the problems they had were family feuds and with fathers of the children. A person would have problems because of the unexpected pregnancy. She would be confused and not know if she should keep the baby or not; she does not know if she will be a good mother or not…* (Counsellor 5)

The counsellors also felt that some participants dropped out because they felt ashamed for sharing sensitive information.

*…Some started, but dropped out after session 1. I think that maybe she thinks or feels that the information she shared with me is too much and cannot face me the following day. ”* (Counsellor 3)

All the counsellors felt that reasons for non-engagement included: i) participants moving from the initial address given at recruitment and not leaving new contact details; ii) participants’ phones getting stolen; and iii) participants living in dangerous or inaccessible areas which resulted in loss of contact. The counsellors indicated that they employed various strategies which were effective in following up participants, such as visiting the participant’s last known address and speaking to neighbours, checking clinic records for updated details and being patient and persistent with following up their participants when they rescheduled appointments. These were in addition to the trial initiated strategies such as giving participants vouchers to compensate them for participating in the study and providing transport to the counsellors for conducting home visits to inaccessible or dangerous areas.

## Discussion

Our findings reflect a wide variation between the counsellors in the rate of dropouts, numbers of sessions attended and fidelity ratings. Together, the six counsellors managed to counsel a total of 156 women out of 209 (74.6%) in sessions which shows reasonable reach despite the challenges encountered. Only 100 (47.8%) of the total number of recruited intervention arm participants (*n* = 209) received at least 6 sessions. This compares reasonably well to the “Thinking Healthy Programme” in Pakistan which had 26% (*n* = 463) receiving the full intervention [22]. The average fidelity rating for all six counsellors was 62.8% which reflects moderate to good fidelity to the manual. The findings however, reflect variation in fidelity measures, attendance and dropout rates. Further statistical analysis would be needed to conclude if there is a correlation between the fidelity scores and attendance rates. Counsellor 1 had the highest number of women who completed the sessions despite having the lowest education level. She had relevant work experience and maturity which is associated with respect in the community [27, 28]. This concurs with the AFFIRM-SA formative study which found that participants preferred counselling by an older woman with practical experience of ‘knowing what she was doing’ [11].

Our findings also reflect positive feedback from all the counsellors who delivered the intervention despite the barriers to implementation. Counsellor facilitators included motivation, empathy and altruism which is consistent with findings from Greenspan and Colleagues [42]. Counsellors were further motivated and became more confident through positive feedback from participants. The counsellors’ health promotion background could have hindered their fidelity to the intervention since health promotion focused on advice giving whereas the intervention required them to be more collaborative by inviting the participants to develop their own solutions to problems. Although some counsellors reported deviating from the manual to explain concepts, they indicated that they enjoyed the sessions and maintained fidelity to the manual since they understood the importance of adhering to the manual. This concurs with Hasson and colleagues who suggest that higher implementation fidelity is assumed when those delivering the intervention are enthusiastic [30].

The results indicate that some counsellors may not have been confident enough to begin counselling sessions and would have preferred a longer training period, however they eventually gained confidence through supervision. Having the supervisor sitting in on some of the early sessions was a way of mitigating the counsellor anxiety and seemed to be received well by some counsellors who attributed their growth to supervision. However, it could have exacerbated the counsellor’s anxiety as indicated by the counsellor who wished the supervisor was not there. More self-reflection questions can be added to the training manual in future to assess counsellor readiness to begin counselling. While their confidence may have been low initially, counsellors also indicated that having a manual, conducting the counselling in their local language, ongoing supervision and training helped to make the counselling easier.

The findings show that the content of the intervention made it easier to deliver the intervention since it was beneficial to both the counsellors and their participants. The most popular sessions among the counsellors were healthy thinking and problem solving which offered practical steps on how the mother can prioritise and solve her problems, and identify unhealthy thoughts to replace them with healthy thoughts.

At times counsellors also experienced difficulties in their personal lives which affected how they coped with their work. Emotional support for counsellors assisted counsellors with maintaining their levels of motivation and preventing burnout [27]. Although the service was available only two counsellors made use of the external counselling while the others spoke to the supervisor. Some counsellors described feeling guilty for not being able to do more for participants who needed material assistance which made it harder to deliver the intervention. Supervision therefore focused on coping strategies and referrals of the participants to social workers and NGOs that could offer material assistance. Counsellors also reported growth by learning how to communicate about their own problems as a result of the intervention which concurs with findings from Jordans and colleagues [43].

Our study demonstrates that attendance rates could have been affected by the mechanisms (how the intervention was received by the participants) [25]. Session 1 was the most attended session, drop out occurred primarily after this session due to several reasons which will be explored in detail in a separate paper focusing on participants’ perspectives of the intervention. It may be possible that women who were distressed due to a crisis, such as an unplanned pregnancy, can benefit from shorter term counselling which focuses only on problem solving and healthy thinking. On the other hand, women with long standing problems, such as recurrent partner infidelity and multiple trauma may benefit from long term counselling and referrals to other organisations. Revising the manual and reducing the number of total sessions from six to three based on the counsellor’s perceptions of the two most helpful sessions would be the next step. This process would also need to be informed by views from the service users. Asking the participant if she feels that her issues have been resolved and would like to either terminate or continue with sessions also gives participants the responsibility to make their own decisions about the duration of the treatment. The use of telephonic counselling and social media could be investigated as other avenues for providing the intervention.

Lastly, counsellors thought older participants seemed more consistent in attendance compared to younger participants. This is similar to the finding by Baron and colleagues [44] on attendance rates for antenatal counselling by trained non-specialist workers. The counsellors experienced a lack of private space to conduct counselling which concurs with findings from Padmanathan and De Silva [27] and counsellors also reported not feeling welcome in clinic A. This is highlighted by the counsellors seeking alternative venues for their sessions and having introduced themselves repeatedly to the nurses. These barriers could have led to the erratic attendance as some participants had to have their sessions outside the clinic. Once the supervisors met with the clinic head this was resolved and a container with partitions was used for the counselling sessions. At times the counsellors wanted to do home visits but were concerned about their safety due to the crime in the community. The counsellors would have to wait for the participants to attend the clinic which affected their delivery of the intervention.

### Implications

This study reveals that process evaluation is necessary to pin point aspects of an intervention which need to be improved through looking at the implementation, mechanisms and context of the intervention for the replication an intervention [25, 29]. The findings also reveal that task sharing is a feasible way of delivering interventions in LAMICS provided there is adequate training and supervision for the lay counsellors. An individual can be trained to become a lay counsellor if they demonstrate the capacity and desire to be empathic and altruistic; however ongoing assessment of empathy and motivation is crucial when recruiting, training and supervising counsellors. Referral to other organisations is necessary when dealing with complex cases that are beyond the scope of the task shared intervention and counsellors should be given information on these additional resources. It is also important to ensure that supervisors of task shared interventions are supported in order to prevent burnout. With additional training and support CHWs can be absorbed as lay counsellors in resource-poor community health and social welfare services in order to increase access to mental health care.

### Limitations

There are several limitations to this study, which need to be noted. While all six counsellors were interviewed, their perspectives would not necessarily be those of a larger group of counsellors. The fidelity rating identified in this study is based on six sessions of only one participant per counsellor. Although a good indication of fidelity, a larger sample size may provide a more consolidated assessment of fidelity. MM was the Mental Health counsellor who trained and supervised the counsellors including assessing for fidelity to the manual, although we do concede the possibility of bias, this was addressed as far as possible by the verification and moderation of the fidelity checks by MS who discussed any discrepancies with MM until consensus was reached. To control for fidelity bias the participants used for the rating were selected randomly. The addition of the perspective from the service users, the focus of a separate paper in preparation, will give a holistic picture of the intervention.

## Conclusion

Task shared interventions can be beneficial for treatment of perinatal depression. Exploring the counsellors’ perspectives provided useful information on facilitators and barriers to the successful implementation of the intervention. Facilitating factors included the content of the intervention (especially problem solving and healthy thinking sessions), ongoing training and supervision, using a counselling manual, conducting counselling sessions in isiXhosa, maintaining fidelity to the manual, counsellors’ motivation and confidence to conduct the sessions, and participant factors such as older age, commitment and a desire to be helped. Barriers included contextual factors such as poverty, crime and lack of space to conduct counselling sessions. Participant factors such as the nature of the participant’s problem, young age, and avoidance behaviour were associated with erratic attendance and drop out of care. Careful attention needs to be paid to ongoing supervision and quality of care if community health workers are to deliver good quality task shared counselling interventions in under-resourced communities.

## Additional files

Additional file 1: Fidelity Checklist. (DOCX 14 kb)

Additional file 2: A Frica Focus on Intervention Research on Mental health (AFFIRM): Post Intervention Semi-Structured Interview schedule for lay counsellors. Full Counsellor Interview schedule used post-intervention for AFFIRM trial. (DOCX 46 kb)

Additional file 3: Consolidated criteria for Reporting Qualitative Research (CORE Q 32. Item checklist). A checklist of 32 items that should be included in qualitative research. (DOCX 15 kb)

## Abbreviations

AFFIRM-SA: AFrica Focus in Intervention Research for Mental health South Africa
CBT: Cognitive behavioural therapy
CHC: Community Health Centre
CHW: Community Health Workers
CPMD: Common perinatal mental disorders
EPDS: Edinburg postnatal depression scale
IPT: Interpersonal therapy
LAMIC: Low and middle income countries
MHC: Mental health counsellor
MRC: Medical research council
NGO: Non-governmental organisation
PST: Problem solving therapy
RCT: Randomised controlled trial
UK: United Kingdom
