# Supplementary material for: A process evaluation exploring the lay counsellor experience of delivering a task shared psycho-social intervention for perinatal depression in Khayelitsha, South Africa
Source: BMC Psychiatry. 2017 Jul 1;17:236. doi: 10.1186/s12888-017-1397-9 (PMC5493861; doi:10.1186/s12888-017-1397-9)
Supplement: Supplementary file 1 — Fidelity Checklist. (DOCX 14 kb) [file 12888_2017_1397_MOESM1_ESM.docx]

**Additional file 1 Fidelity Checklist**

Name of counsellor: ------------------- PID of participant: ---------------------------- Session Number: --------------- Topic for the session: -------------

| **Description** | Not done [0] | Needs Improvement [1] | Well done [2] | Not applicable [99] | Comments |
| --- | --- | --- | --- | --- | --- |
| **Step 1: Introduction** | | | | | |
| 1. Build rapport (small talk) |  |  |  |  |  |
| 1. Clarifies issues for discussion |  |  |  |  |  |
| **Step 2: Exploration** | | | | | |
|  | Not done [0] | Needs Improvement [1] | Well done [2] | Not applicable [99] | Comments |
| 1. Explores use of relaxation CD and activity book |  |  |  |  |  |
| 1. Introduces topic to be explored and explains exercise clearly |  |  |  |  |  |
| 1. Active listening and probing |  |  |  |  |  |
| 1. Clarifies the participant’s concerns |  |  |  |  |  |
| 1. Empathy and non-judgemental attitude |  |  |  |  |  |
| 1. Practices exercise with participant and encourages healthy behaviour |  |  |  |  |  |
| **Step 3: Ending** | | | | | |
|  | Not done [0] | Needs Improvement [1] | Well done [2] | Not applicable [99] | Comments |
| 1. Summarises and clarifies the main issues |  |  |  |  |  |
| 1. Gives clear instructions for homework and checks if participant understands |  |  |  |  |  |
| **TOTAL** |  |  |  |  |  |

**Additional Notes:**
