# Supplementary material for: A process evaluation exploring the lay counsellor experience of delivering a task shared psycho-social intervention for perinatal depression in Khayelitsha, South Africa
Source: BMC Psychiatry. 2017 Jul 1;17:236. doi: 10.1186/s12888-017-1397-9 (PMC5493861; doi:10.1186/s12888-017-1397-9)
Supplement: Supplementary file 2 — A Frica Focus on Intervention Research on Mental health (AFFIRM): Post Intervention Semi-Structured Interview schedule for lay counsellors. Full Counsellor Interview schedule used post-intervention for AFFIRM trial. (DOCX 46 kb) [file 12888_2017_1397_MOESM2_ESM.docx]

**Supplementary File 1**

**AFrica Focus on Intervention Research on Mental health (AFFIRM): Post Intervention Semi-Structured Interview schedule for lay counsellors**

**Instructions**

*Probes - nantsi imizekelo ngezantsi:*

- Ask for examples to explain what she says
- *Cela ukuba akunike imizekelo ukucacisa oko akutshoyo.*
- ‘Tell me more’.
- *Ndixelele ngakumbi*
- That’s really useful to know. Please tell me more and explain further.
- *Kubalulekile ukwazi. Ndicela undixelele ngakumbi undicacisele ngakumbi.*

**Participant Name: __________________________________**

START:

Thank you for making the time to talk to us today. The purpose of talking to you is to find what you think about working on the AFFIRM study. Please remember that you will not be judged for anything you say in this interview therefore I would like you to be as honest as possible. **This interview is not a counselling session, it is for us to find out your opinion so that we can improve our programme.**

*Siyabulela ngokuthi wenze ixesha lokuthetha nathi namhlanje. Eyona njongo yale ncoko yanamhlanje kukufumanisa ukuba uqhuba njani ukusukela oko wavuma ukuthabatha inxaxheba kuphando lwethu. Nceda ukhumbule ukuba akukho mntu uzokugweba ngento oyithethe kolu ndliwano ndlebe ngoko ndingathanda ukuba unyaniseke kangangoko unako. Oludliwano ndlebe ayiyo iseshinsi yokunika inkxaso, ludliwano ndlebe lokubuza indlela ocinga malunga neziseshini owazifumanayo.*

**1. Capacity building**

1. What is your reason for wanting to be a counsellor?

*Yintoni isizathu sokuba ufune ukuba ngumcebisi?*

1. What do you think about the week of training that you got before you started counselling with AFFIRM? Was it long enough? Do you think that it trained you enough to be able to do the counselling properly?

Ucinga ntoni ngala veki yokuqeqeshwa wayifumana phambi kokuba uqale iseshini zeengcebiso neAFFIRM? Ingaba yayilixesha elaneleyo na? Waqeqeshwa ngokwaneleyo ukwenzela ukuba ube ngumcebisi ogqibeleleyo?

c. Can you describe your feelings and your story about how things changed from doing the training and when you actually started doing counselling sessions with participants? What was that change like for you, and how did you manage it?

Ungachaza imizwa yakho nebali lakho ngedlela izinto zatshintsha ngayo ukusukela ngoku wawuqeqeshwa ukuzotsho ukuqala kwakho ukwenza iseshini zengcebiso.

d. What improvements can you recommend for the training for counsellors?

*Ngeziphi iingcebiso ongazitsho ukuphucula ukuqeqeshwa kwabacebisi kwixesha elizayo?*

**2. Environment Issues**

1. How did you manage to do counselling sessions in the clinics? For example, speaking to the nurses, getting space to have the sessions?

*Ukwazi njani ukwenza iseshini zengcebiso kwikliniki. Umzekelo, ukuthetha nabongikazi ukufuna indawo yokusebenzela wenze iseshini yengcebiso?*

1. How do you think other nurses and clinic staff accepted you at the clinic? Did you have to do any negotiating to be able to work there? If so, what did you do? If you could rate their acceptance of you there, between 0 and 10, what number would you give it? 0 (you weren’t accepted) -10 (you were very accepted)?

*Ucinga ukuba abanye abongikazi nabantu abasebenza ekliniki bakwamkele njani? Ukhe kwafuneka wenze uthetho thethwano? Ukuba kunjalo wenze ntoni? Ukuba ungamentesha indlela abakwamkele ngayo ungathini ukususela ku0 uyotsho ku10 mgeliphi inani onokulinika? 0 (wawungamkelekanga) – 10 (wawuwamkelekile khakhulu)*

1. How did you make contact with the clients to start the counselling?

*Wenze njani ukuqhakamshalena nomxhaswa ukuze niqale iseshini zengcebiso?*

1. How many clients did you have in total during the course of the trial?

*Bangaphi abaxhaswa oyewanabo ekugqibeleni?*

**3. Self-Assessment**

1. Before you started the sessions what were your fears?

*Phambi kokuba uqale iiseshini yintoni wawuyoyika?*

1. How did you rate yourself as a counsellor before you started working on AFFIRM? On a scale of 0 to 10. (Please explain)

*Wazimentesha njani njengomcebisi phambi kokuba uqale ukusebsenzela iAFFIRM. Uqala ku0-10 (ndicela ucacise)*

1. How would you rate yourself as a counsellor now? On a scale of 0 to 10. *(Please explain)*

*Ungazimentesha uthini ngoku njengomcebisi ukuqala ku 0-10 (ndicela ucacise)*

1. How was your first session? Were any of these fears confirmed or removed after this session?

*Kwakunjani ekuqaleni kwakho ukuba neseshini? Ingaba uvalo wawunalo lwaluyinto yamanyani okanye lwasuka emveni ugqibile iseshini yokuqala?*

1. What did you find easy when delivering the intervention? Or what made it easy to deliver the intervention?

*Yintoni eyenze kube lula ukucebisa okanye yintoni ibilula xa ubungumcebisi?*

1. What are your thoughts on the counselling being conducted in Xhosa?

*Ucinge ntoni njengoba iiseshini zengcebiso zingesiXhosa?*

**4. Challenges Faced**

1. What are the challenges that you faced in delivering the counselling sessions? What things made it more difficult to do the counselling in the way that you had been trained to do it?

*Ngeziphi iingxaki ubenazo xa ubungumcebisi? Ngeziphi izinto ezenze ukucebisa kwanzima akwabikho njengohlobo obuqeqeshwe ngalo?*

1. What can be done to make these challenges easier?

*Yintoni enokwenziwa ukuze ziphele ezingxaki?*

1. What helped you or made things easier for you to do the counselling?

Yintoni ibiluncedo okanye yenza kwalula ukwenza iseshini yengcebiso?

1. What other general kinds of things do you think might have made the counselling difficult for you? For example, money problems, crime, taxi strikes, political issues, or social problems?

*Ngezipho ezinye izinto ezinokuba sisiqobo xa ucinga ekwenzeni ingcebiso? Umzekelo ingxaki zemali, ubukrelemqa, uqhushululu lono teksi, ezezopolitiko, okanye imeko yasekuhlaleni?*

1. Did you have any sessions in which your clients asked for help with money or practical support? How did you manage that situation? How did your client respond?

Ukhe wabeneseshini apho umxhaswa ebeku cele uncedo lwemali okanye inkxaso nokuba ngoluphi luhlobo? Uye wathini kulo meko? Umxhaswa yena uye wathini?

**5. Attendance**

1. What would you say the difference is between working with younger or older clients? Is there a difference in their attendance? Describe your experience of working with younger or older clients in terms of overall attendance.

*Yintoni umehluko phakathi kokusebenza nabantu abatsha okanye abantu abadala? Ukhona umehluko kwindlela abazihamba ngayo iiseshini? Kha undichazele ngolwakho uluvo phakathi kosebenza nabantu abatsha okanye abadala nendlela abahamba ngayo iseshini zengcebiso?*

1. How many of your clients had all 6 sessions?

*Bangaphi kuba xhaswa bakho abaye bazihamba zonke zontandathu iseshini zengcebiso.*

1. How many sessions did you manage to have on average with each client? What was the highest session number attended?

*Zingaphi iiseshini oye wakwazi ukuba nazo nomxhaswa ngamnye? Ngeliphi inani elikhulu kwiseshini uye walifumana.*

d. What did you notice about the type of clients who were good at coming to sessions and the type of clients who didn’t come? What were those different clients like? E.g. older or younger clients for those that attended all 6 sessions what do you think led them to attend all 6 or what kept them coming for sessions?

*Yintoni oyiqapheleyo ngodidi lwabaxhaswa ababe khuthele ukuhamba iiseshini zengcebiso kwakunye nabo bangakhange beze? Bebenjani? Bebe badala okanye bebe batsha. Xa ucinga yintoni isizathu sabo sokunga hambi iiseshini zengcebiso. Yaye abaye baza kuzo zosithandathu yintoni ibibanika umdla wokuba bemanye besiza onke amaxesha.*

*e.* How many people stopped attending sessions?

*Bangaphi abaye bayeka ukuhamba iiseshini zengcebiso kwaphela?*

f. What reasons did they give for no longer attending the sessions?

*Ngeziphi izizathu abaye baziniki xa babeyeka?*

g. How would they show you that they were no longer interested in attending the sessions?

*Babezibonakalisa njani ukuba abasanamdla wokuhamba isheshini zengcebiso?*

h. What other general kinds of things do you think might have made it difficult for the clients to attend the counselling? For example, money problems, crime, taxi strikes, political issues, or social problems?

Ngeziphi ezinye izizathu ezenze kwanzima ukuba abaxhaswa bahambe iiseshini zengcebiso. Umzekelo, ingxaki zemali, ubukrelemqa, uqhushululu lonoteksi, ezoplitiko okanye imeko yase kuhlaleni?

i. How many clients did not attend any sessions? What were their reasons for non-attendance?

*Bangaphi kuba xhaswa bakho abanga khange beze kwiseshini zengcebiso. Ibiyintoni isizathu sabo sokungazi kwiseshini zengcebiso?*

j. Describe the progress of the clients in terms of being better or worse after receiving the counselling? Were there any clients who got worse after the counselling?

*Ndicela undichazele inqubela yabaxhaswa malunga nokuba ngcono okanye ibengakumbi emveni kokuba befumene uncedo, ingaba bakhona abantu abanga ncedekanga kukuhamba iiseshini?*

k. Describe your relationship with your clients? Did you change your counselling style for different clients? Can you explain about this?

*Ndicela undichazele ubuhlobo obunabo kunye nabaxhaswa bakho. Uye watshintsha indlela ocebisa ngayo na kubanye abantu okanye ibifana kubo bonke abantu? Ungandicacisela ngakumbi?*

**6. Fidelity**

1. Were you able to use the manual as intended?

*Ubukwazi ukusebenzisa incwadi yemanyuwali njongokuba injalo?*

1. Were you always able to conduct sessions in the way it said in the manual? If not what was the reason for changing the session?

*Ubukwazi ukuziqhuba iiseshini zengcebiso ngale ndlela zibekwe ngayo? Ukuba hayi sithini isizathu esikwenze watshintsha iiseshini?*

1. How did you make referrals and collaborate with other service providers?

*Yintoni ebikwenza ufune ukuthumela umntu ayekufuna uncedo yaye ubuqhakamshelana njani nabanye abancedisi bezinye iinkonzo?*

1. How would you explain the different session topics:

*Ungachaza njani lamanqaku alandelayo ebekwi seshini*

- Psycho-education about depression
- *Ukufunda nongxunguphalo*
- Problem solving
- *Ukusombulula Ingxaki*
- Behaviour Activation
- *Imo yokuziphatha*
- Healthy thinking
- *Ingcinga ezizo*
- Psycho-education for birth preparation
- *Ukulungiselelea ukubeleka*
- Termination and evaluation
- *Uvavanyo noPhononongo*

**7. Effectiveness of the intervention**

- 1. What do you think was the most effective part of the counselling (the part that helped the mothers most)?

*Ngokwengcinga zakho ngeyiphi eyona ngcebiso ibiqotho okanye ibuluncedo kwiseshini eyona incede oomama abaninzi?*

- 1. Were there particular sessions that you think were most helpful? Which *sessions were these?*

*Zikhona iiseshini owuthi xa ucinga ubone ukuba bezuluncedo? Ngeziphi ezoseshini?*

- 1. Was there anything particular that you did that helped the mothers to feel more comfortable in the counselling? (If they need examples: e.g. listening, not judging, giving advice, providing a safe confidential place for the mothers to talk, etc.)?

*(This question is intended to explore what factors in the counsellor’s personal style or relationship with the clients were most helpful).*

*Ikhona into oyenzileyo eyeyanceda oomama umzekelo ukumamela, ukungagwebi, ungamniki ngcebiso okanye unike ingcebiso, ukwenza indawo ekhuselekileyo ukwenzela oomama bakhuleleke xa befuna ukuzikhuphela imbilini zabo etc?*

*Lo mbuzo wenzelwe ukuba ukwazi ukuphulisa ngeziphi izinto bezuluncedo kwindlela umcebisi ebecebisa ngayo okanye nendlela yokwakha ubuhlobo nabaxhaswa ibuluncedo.*

d. Were there any particular sessions, or any particular things that you did that you thought afterwards were not very helpful? If so, what were these?

*Zikhona iiseshini okanye izinto oye wazenza apho uyewaqonda ukuba khange zincede? Ukuba ewe ngeziphi?*

**8. Motivation for sessions**

1. Which was your favourite session and why?

*Ngeyiphi iseshini oyewayonwabela? (nceda ucacise)*

1. Which was your worst session and why?

*Ngeyiphi ongakhange uyonwabele? (nceda ucacise)*

1. What could you have done differently in the sessions?

*Ingaba ikhona enye into eyahlukileyo ongeyenzile?*

d*.* Do you have any suggestions for ways of improving the way that the 6 sessions work?

*Ingaba unazo ingcebiso nendlela apho kungaphuculwa imeko yeseshini zosithandathu?*

e. Do you feel that you have changed as a counsellor? If yes, are the changes positive or negative?

*Uziva utshintshile njengomcebisi? Ukuba ewe, ingaba lutshintsho olubi okanye oluhle?*

1. Would you recommend that others do this work?

*Ungafuna abanye abantu bawenze lomsebenzi uwenzayo?*

We are now finished with the questions. Do you have any questions or anything else you want to say about this interview?

*Sigqibile ngoku ngemibuzo. Ingaba unayo imibuzo okanye nokuba yintoni ofuna ukuyithetha ngolu dliwano-ndlebe?*

***Thank you for participating in our study!***

***Enkosi ngokuthatha inxaxheba kuphando lwethu!***
