# Supplementary material for: A process evaluation exploring the lay counsellor experience of delivering a task shared psycho-social intervention for perinatal depression in Khayelitsha, South Africa
Source: BMC Psychiatry. 2017 Jul 1;17:236. doi: 10.1186/s12888-017-1397-9 (PMC5493861; doi:10.1186/s12888-017-1397-9)
Supplement: Supplementary file 3 — Consolidated criteria for Reporting Qualitative Research (CORE Q 32. Item checklist). A checklist of 32 items that should be included in qualitative research. (DOCX 15 kb) [file 12888_2017_1397_MOESM3_ESM.docx]

**Supplementary file 2**

**Consolidated criteria for Reporting Qualitative Research (CORE Q 32. Item checklist)**

**Number Item Guide Questions/ Description**

**Domain 1: Research Team and Reflexivity**

Personal Characteristics

1. Interviewer Which author conducted the interview?

None of the authors conducted the interviews. A trained field worker, Pahliwe Memani conducted the interviews.

1. Credentials What were the researcher’s credentials?

The field worker has a grade 12 certificate (High school qualification) and a Diploma in public administration. The field worker has undergone extensive training on qualitative research methods as part of the study.

1. Occupation What was their occupation at the time of the study?

Pahliwe Memani was a field worker who was trained to conduct interviews for the control group during the randomized control trial and then trained to conduct post intervention qualitative interviews after the conclusion of the intervention.

1. Gender Was the researcher male or female?

The researcher was female.

1. Experience and training What experience and training did the researcher have?

The researcher was trained on qualitative research, and how to conduct the telephonic follow-up questions for the AFFIRM RCT control group between May 2013 and May 2016.

1. Relationship established? Was a relationship established prior to study commencement?

No, a relationship was not established prior to study commencement. This was to avoid familiarity that could affect the participant’s freedom to talk during the interview.

1. Participant knowledge of the interviewer What did participant know about the researcher?

Participants knew that the interviewers was a field worker for AFFIRM who conducted telephone calls with the control participants prior to the interviews.

1. Interviewer characteristics What characteristics were reported about the interviewer?

The interviewer was interested in the interviews as she was already part of the AFFIRM team, bias was controlled for by providing her with training.

**Domain 2: Study Design**

1. Methodological orientation and theory What methodological orientation was stated to underpin the study?

Content analysis was used to underpin the study.

Participant selection

1. Sampling How were participants selected?

There was no sampling as all the lay counsellors from the AFFIRM study were included.

1. Method of approach How were participants approached?

Participants were approached face-to-face.

1. Sample size How many participant were in the study?

6 counsellors were selected and 6 participants (1 per counsellor were selected for fidelity checking of 6 counselling sessions).

1. Non-participation How many people refused to participant or dropped out? Reasons?

No participants refused to participate or dropped out.

Setting

1. Setting of data collection Where was the data collected?

The data was collected at the clinic

1. Presence of non-participants Was anyone else present besides the participants and researchers?

No one else was present besides participants and researchers.

1. Description of sample What are the important characteristics of the sample?

All the participants were female counsellors who had delivered a task shared intervention for perinatal depression. They all had at least 2 and a half years of health promotion experience amongst themselves. Their ages were 28, 32, 33, 40, 44 and 46. Please refer to table 2 for a profile of respondents.

Data Collection

1. Interview guide Were questions, prompts, guides provided by the authors? Was it pilot tested?

Questions, prompts and guides which were developed by the AFFIRM team were provided and piloted in order to test and correct any errors.

1. Repeat interviews Were repeat interviews carried out? If so how many?

One repeat interview was carried for a counsellor due to the battery going flat in the middle of the interview.

1. Audio/Visual recording Did the research use audio visual recording to collect the data?

Yes, the research used digital audio recorders to collect the data.

1. Field notes Were field notes made during and or after the interview or focus group?

No field notes were not made during the interviews.

1. Duration What was the duration of the interviews or focus groups?

The interviews lasted between 40 to 61 minutes per counsellor.

1. Data Saturation Was data saturation discussed?

No, data saturation was not discussed as the full population of 6 counsellors participated.

1. Transcripts returned Were transcripts returned to participants for comment and or correction?

No transcripts were not returned to participants for comment and or correction as they had already been invited to provide additional comments during the interview stage. There are various methodological and ethical questions that arise due to the member-check strategy, although it can increase credibility, it is quite complex and it is difficult to predict how the participants will experience the gesture. Member-check therefore presents a topic which needs further research.

**Domain 3: Analysis and findings**

Data Analysis

1. Number of data coders How many data coders coded the data? MM coded the interview transcripts and MS reviewed the themes and discussed with MM if there was any query. MM and MS coded the counselling session transcripts for fidelity coding independently and discussed if there were any queries.
2. Description of coding tree Did authors provide a description of the coding tree?

No, the authors did not provide a description of the coding tree.

1. Deviation of themes Were themes identified in advance or derived from data?

Some broad themes were identified in advance after conducting the literature search and some were derived from the data all themes were integrated once coding had been completed.

1. Software What software if applicable, was used to manage data?

NVivo v11 was used to manage the data.

1. Participant checking Did participant provide feedback on the findings?

No, participants did not provide feedback on the findings.

1. Quotations presented Were participant quotations presented to illustrate the themes/ findings? Was each quotation identified?

Yes, participant quotations were presented to illustrate the themes/ findings and each quote was identified.

1. Data and findings consistent Was there consistency between the data presented and the findings?

Yes, there was consistency between the data presented and the findings.

1. Clarify the major themes Were major themes clearly presented in the findings?

Yes, major themes were clearly presented in the findings.

1. Clarity of minor themes Is there a descriptions of diverse cases or discussion of minor themes?

Yes, there is a discussion of minor themes.
